# Incidence, predictors and patterns of care of patients with very severe hypertriglyceridemia in Ontario, Canada: a population-based cohort study

**DOI:** 10.1186/s12944-021-01517-6

**Published:** 2021-09-03

**Authors:** Amanda J. Berberich, Alexandra M. Ouédraogo, Salimah Z. Shariff, Robert A. Hegele, Kristin K. Clemens

**Affiliations:** 1 Department of Medicine and Robarts Research Institute, Schulich School of Medicine and Dentistry, Western University, London, Ontario N6A 5B7 Canada; 2 St. Joseph’s Health Care London, 268 Grosvenor St, London, ON N6A 4V2 Canada; 3 ICES, London, Ontario Canada; 4 Lawson Health Research Institute, London, Ontario Canada; 5 Departments of Medicine and Epidemiology and Biostatistics, Western University, London, Ontario N6A 5B7 Canada

**Keywords:** Triglycerides, Pancreatitis, Fibrates, Secondary hypertriglyceridemia

## Abstract

**Background:**

The incidence of severe (S-HTG) and very severe hypertriglyceridemia (VS-HTG) among Canadians is unknown. This study aimed to determine the incidence, characteristics, predictors and care patterns for individuals with VS-HTG.

**Methods:**

Using linked administrative healthcare databases, a population-based cohort study of Ontario adults was conducted to determine incidence of new onset S-HTG (serum triglycerides (TG) > 10–20 mmol/L) and VS-HTG (TG > 20 mmol/L) between 2010 and 2015. Socio-demographic and clinical characteristics of those with VS-HTG were compared to those who had no measured TG value > 3 mmol/L. Univariable and multivariable logistic regression were used to determine predictors for VS-HTG. Healthcare patterns were evaluated for 2 years following first incidence of TG > 20 mmol/L.

**Results:**

Incidence of S-HTG and VS-HTG in Ontario was 0.16 and 0.027% among 10,766,770 adults ≥18 years and 0.25 and 0.041% among 7,040,865 adults with at least one measured TG, respectively. Predictors of VS-HTG included younger age [odds ratios (OR) 0.64/decade, 95% confidence intervals (CI) 0.62–0.66], male sex (OR 3.83; 95% CI 3.5–4.1), diabetes (OR 5.38; 95% CI 4.93–5.88), hypertension (OR 1.69; 95% CI 1.54–1.86), chronic liver disease (OR 1.71; 95% CI 1.48–1.97), alcohol abuse (OR 2.47; 95% CI 1.90–3.19), obesity (OR 1.49; 95% CI 1.13–1.98), and chronic kidney disease (OR 1.39; 95% CI 1.19–1.63).

**Conclusion:**

The 5-year incidence of S-HTG and VS-HTG in Canadian adults was 1 in 400 and 1 in 2500, respectively. Males, those with diabetes, obese individuals and those with alcohol abuse are at highest risk for VS-HTG and may benefit from increased surveillance.

**Supplementary Information:**

The online version contains supplementary material available at 10.1186/s12944-021-01517-6.

## Background

Triglyceride (TG) values > 20 mmol/L can have significant consequences. One of the most detrimental outcomes is pancreatitis, which often requires hospitalization or admission to intensive care, and associated mortality. Pancreatitis is linked with the presence of chylomicrons in the serum, which occurs when TG levels exceed 10 mmol/L, with risk rising sharply as levels exceed 20 mmol/L [1–5]. In clinical practice, very severe hypertriglyceridemia (VS-HTG), defined in this study as triglyceride (TG) levels that exceed 20 mmol/L, is captured through routine lipid testing and commonly misattributed to an exclusive genetic etiology. A minority of cases of VS-HTG (~ 1 in 1 million) arise from a primary TG disorder, typically caused by bi-allelic mutations in genes including lipoprotein lipase (*LPL*), apolipoprotein C-II (*APOC2*), apolipoprotein A-V (*APOA5*), lipase maturation factor 1 (*LMF1*) and glycosylphosphatidylinositol-anchored high density lipoprotein binding protein 1 (*GPIHBP1*) [6]. Most cases result from secondary causes, often in conjunction with inherited partial impairment in TG metabolism [7–11]. These cases may only manifest under conditions that increase TG production or impair clearance. Previous studies conducted elsewhere have suggested that secondary causes of VS-HTG, include obesity and metabolic syndrome, poorly controlled diabetes, nephrotic syndrome, severe hypothyroidism, oral estrogen or tamoxifen, glucocorticoids, beta blockers, retinoids and HIV antiretroviral regimens [3, 12, 13]. Irrespective of the underlying cause, VS-HTG is of clinical concern due to the risk of HTG-associated pancreatitis. Thus, it remains important to understand and manage the secondary risk factors associated with this condition [5].

While studies conducted in other regions, such as Norway and the United States, have suggested the prevalence of S-HTG to be between 0.13–0.4% [14, 15] and VS-HTG to be 0.05–0.1% [16, 17], there is minimal documentation of the epidemiology of S-HTG or VS-HTG in Canada. This study examined the incidence of S-HTG and VS-HTG in Canada’s most populous province (Ontario), which is ethnically and socially distinct from populations studied previously [14, 16]. The demographic distribution, laboratory features and co-morbidities associated with VS-HTG in Canada were also examined. Further, as studies conducted in regions without universal health care coverage [16] suggest a significant care gap for patients with VS-HTG, the care patterns of Ontario residents with VS-HTG were examined to determine whether a similar care gaps exist.

## Methods

### Design and setting

This was a population-based retrospective cohort study of adults ≥18 years in Ontario, Canada between 2010 and 2015. Ontario’s approximately 13.5 million residents have universal healthcare through the Ontario Health Insurance Program (OHIP), with comprehensive medication coverage provided to those ≥65 years or using social assistance. Information on the use of these health services is maintained at ICES (formerly The Institute for Clinical Evaluative Sciences). ICES is an independent, non-profit research institute whose legal status under Ontario’s health information privacy law allows it to collect and analyze health care and demographic data, for health system evaluation and improvement.

### Participants

All individuals aged ≥18 years with at least one TG value in the Ontario Laboratories Information System (OLIS) between September 1, 2010 and September 1, 2015 were included. This study period was selected to allow for a 2-year follow up for care patterns and outcomes of interest following identification of an incident case (OLIS laboratory data available until the end of 2017). Standard data cleaning excluded those with invalid health card numbers, age ≥ 105 years, missing sex, and non-Ontario residents. Three patient cohorts were then created. Individuals who had TG levels > 20 mmol/L were included in the VS-HTG cohort (cohort 1) and individuals who had TG levels > 10–20 mmol/L were included in the S-HTG cohort (cohort 2). Those with prior evidence of TG > 20 or > 10 mmol/L between September 12, 2007–August 31, 2010 were excluded to define new evidence of VS-HTG or S-HTG respectively.

**Fig. 1:**
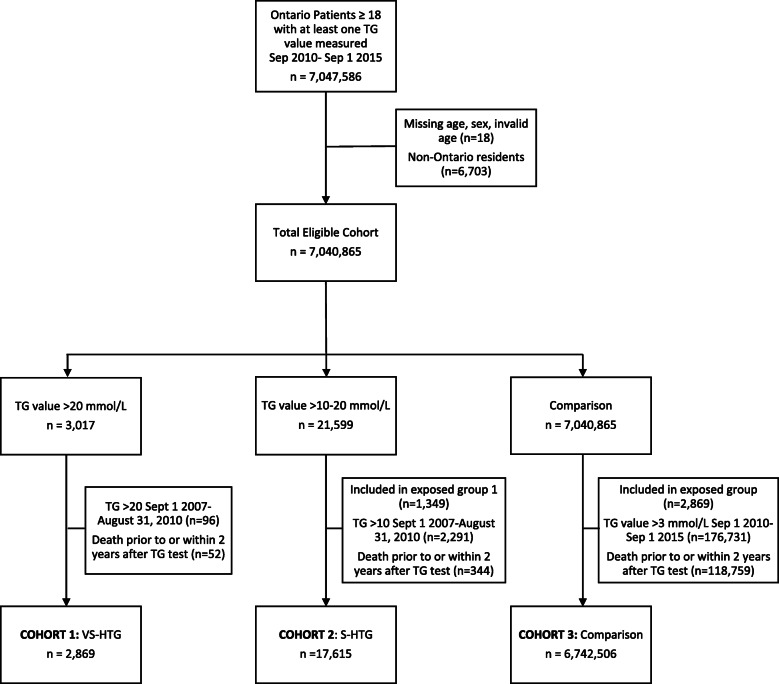
Flowchart of study participants. After removal of duplicate and invalid data, the final cohorts included 2869 individuals in VS-HTG cohort 1 (TG > 20 mmol/L), 17,615 in S-HTG cohort 2 (TG > 10-20 mmol/L) and 6,742,506 in the comparison cohort 3 (no TG > 3.0 mmol/L)

A third cohort (cohort 3) was used to contrast characteristics of those with VS-HTG and with no HTG and establish predictors for VS-HTG. This comparator group included all Ontario residents ages ≥18 years with at least one TG value in OLIS during the study period, no evidence of TG value > 3 mmol/L and who were not already in the S-HTG or VS-HTG cohort. In all cohorts, those who died within 2 years after a TG test were excluded to establish their follow up care patterns. If individuals had more than one TG test during the study period, the first TG test was selected (Fig. 1).

### Sources of data

Data were drawn from a number of ICES databases (Supplemental Table 1). Datasets were linked using unique encoded identifiers and analyzed at ICES. Demographic data were obtained from the Registered Persons Database (RPDB), which includes all individuals who have been issued an Ontario health card. The Client Agency Program Enrolment (CAPE) database was used to determine patients who were rostered to a family physician. The Canadian Institutes for Health Information’s Discharge Abstract Database (CIHI-DAD) and the National Ambulatory Care Reporting System (NACRS) contain diagnostic and procedural information captured during hospitalizations and emergency department visits, respectively, using International Classification of Diseases 10th Revision (ICD-10) [18] and Canadian Classification of Health Interventions (CCI) [19] codes. Additional comorbidities and healthcare services use was captured through OHIP, which contains billing and diagnostic codes (Supplemental Table 1). Hypertension and diabetes status were determined from the hypertension database (HYPER) [20] and Ontario Diabetes Database (ODD) [21], respectively. For individuals ≥65 years, use of medications that can be associated with or used to treat HTG was captured using the Ontario Drug Benefit (ODB) and Drug Identification Number (DIN) database. Laboratory data were obtained through OLIS. The OLIS database holds laboratory data from both community and hospital laboratories with good catchment across Ontario [22]. As of 2016, 95% of community lab volume and over 80% of total provincial lab volume were recorded in OLIS [23]. Supplementary Table 1 includes full details on variable definitions.

### Incidence of VS-HTG

The primary aim was to calculate the incidence of S-HTG and VS-HTG over the 5 year period. This was done using two denominators: the mean number of Ontarians ≥18 years between 2010 and 2015, and the number of adults ≥18 years who had a measured TG level over the study period.

### Outcomes

The 2-year patterns of care of those with VS-HTG were examined, including contacts with family physicians, internists and endocrinologists, repeat TG testing, new prescriptions for fibrates, statins and niacin, and minimum and last follow-up TG values. The secondary exploratory outcomes of interest included hospital encounters for pancreatitis, acute myocardial infarction or ischemic stroke over the 2-year follow up period.

### Statistical analysis

The incidence of S-HTG and VS-HTG was provided as a rate per 100,000. Descriptive statistics were used to capture baseline differences between the VS-HTG cohort and the comparator cohort 3. Continuous variables were reported as mean +/− standard deviation (SD) and median with interquartile range (IQR). Binary variables were reported as percentages. Differences between cohorts were evaluated using standardized differences (StDiff), with a value of > 0.1 considered significant [24]. One-way ANOVA or Chi Square tests were also used to compare the means and medians of continuous variables and the proportions of categorical variables, respectively. *P*-value s < 0.05 were considered statistically significant. Variables that were hypothesized to affect TG levels, co-exist with HTG or related complications from HTG were included in the analysis. To identify predictors of VS-HTG, both univariable and multivariable logistic regression were used (nominal *P-*value for significance < 0.05) and reported results as odds ratios (OR) and 95% confidence intervals (CI). Descriptive statistics were used to report crude rates of secondary outcomes of interest.

### Ethics approval

The use of data in this project was authorized under section 45 of Ontario’s Personal Health Information Protection Act, which does not require review by a Research Ethics Board. Guidelines for reporting of studies outlined by routine collected healthcare data (RECORD) were used (Supplemental Table 2) [25], as well as STROBE cohort reporting guidelines for observational studies [26].

## Results

There were 22,745,387 TG tests performed in Ontario between 2010 and 2015 in those ≥18 years (7,047,586 unique individuals). A total of 17,615 and 2869 people had S-THG and VS-HTG respectively. The comparison cohort included 6,742,506 individuals (Fig. 1).

Baseline differences considered between the VS-HTG cohort 1 and the comparison cohort 3 are shown in Table 1. In general, those with VS-HTG were more often male (78.0% vs 45.6%; StDiff 0.71, *P-*value < 0.001), had diabetes (44.0% vs 13.9%; StDiff 0.7, *P-*value < 0.001), chronic liver disease (7.9% vs 3.4%; StDiff 0.19, *P-*value < 0.001), alcohol abuse (2.3% vs 0.4%; StDiff 0.17; *P*-value < 0.001), obesity (1.8% vs 0.6%; StDiff 0.12; *P-*value < 0.001) and HbA1c > 8.5% (13.8% vs 1.5%; StDiff 0.48; *P*-value < 0.001). Significantly more individuals in the VS-HTG cohort had a baseline history of pancreatitis (6.2% vs 0.3%; StDiff 0.34; *P*-value < 0.001) (Table 1). All measured components of metabolic syndrome appeared higher in the VS-HTG cohort: (HTN (43.4% vs 33.1%; StDiff 0.21; *P*-value < 0.001), low HDL cholesterol (C) (mean 0.97 vs 1.27 mmol/L; StDiff 0.79; *P*-value < 0.001), elevated HbA1c (mean 7.88% vs 6.63%; StDiff 0.67; *P*-value < 0.001) and obesity (1.8% vs 0.6%; StDiff 0.12; *P-*value < 0.001).

**Table 1:** Baseline characteristics of VS-HTG and comparison cohorts

	VS-HTG Cohort ***N*** = 2869	Comparison Cohort ***N*** = 6,742,506	Standardized differences	***P***-value
Age at index date, years				
Mean (SD)	47.28 ± 10.87	51.74 ± 16.21	0.32	< 0.001
Median (IQR)	47.0 (40.00–54.00)	52.0 (40.00–63.00)	0.35	< 0.001
18–30 yrs	174 (6.1%)	744,062 (11.0%)	0.18	< 0.001
31–45 yrs	1078 (37.6%)	1,642,193 (24.4%)	0.29	
46–65 yrs	1481 (51.6%)	2,963,456 (44.0%)	0.15	
66+ yrs	136 (4.7%)	1,392,795 (20.7%)	0.49	
Sex, female	630 (22.0%)	3,667,595 (54.4%)	0.71	< 0.001
Income quintile^a^				
1 - lowest	690 (24.1%)	1,231,411 (18.3%)	0.14	< 0.001
2	649 (22.6%)	1,323,360 (19.6%)	0.07	
3	635 (22.1%)	1,371,761 (20.3%)	0.04	
4	493 (17.2%)	1,432,535 (21.2%)	0.1	
5 - highest	402 (14.0%)	1,383,439 (20.5%)	0.17	
Rostered to a GP	2346 (81.8%)	5,619,709 (83.3%)	0.04	0.023
**Comorbidities**				
Diabetes (Type 1 and Type 2)	1262 (44.0%)	940,151 (13.9%)	0.70	< 0.001
Hypertension	1244 (43.4%)	2,232,885 (33.1%)	0.21	< 0.001
Coronary artery disease (excluding angina)	339 (11.8%)	600,499 (8.9%)	0.10	< 0.001
Cardiovascular disease	87 (3.0%)	218,670 (3.2%)	0.01	0.524
Peripheral vascular disease	18 (0.6%)	21,539 (0.3%)	0.04	0.003
Chronic liver disease	226 (7.9%)	230,046 (3.4%)	0.19	< 0.001
Alcohol	66 (2.3%)	24,957 (0.4%)	0.17	< 0.001
Obesity	53 (1.8%)	38,645 (0.6%)	0.12	< 0.001
Hypothyroidism	15 (0.5%)	24,618 (0.4%)	0.02	0.161
Multiple myeloma	0	1060 (0.0%)	0.02	0.502
Nephrotic syndrome	29 (1.0%)	14,392 (0.2%)	0.10	< 0.001
Chronic kidney disease	199 (6.9%)	209,348 (3.1%)	0.18	< 0.001
Acute Pancreatitis	177 (6.2%)	19,602 (0.3%)	0.34	< 0.001
Gallstone disease	212 (7.4%)	114,035 (1.7%)	0.28	< 0.001
Current pregnancy in women	47 (1.6%)	385,120 (5.7%)	0.22	< 0.001
Charlson comorbidity index				
Mean (SD)	0.92 ± 1.38	0.44 ± 1.05	0.39	< 0.001
Median (IQR)	0.00 (0.00–1.00)	0.00 (0.00–0.00)	0.52	< 0.001
0	2241 (78.1%)	6,104,946 (90.5%)	0.35	< 0.001
1	331 (11.5%)	302,652 (4.5%)	0.26	
2	142 (4.9%)	193,468 (2.9%)	0.11	
3+	155 (5.4%)	141,440 (2.1%)	0.17	
**Health care utilization**				
Primary care (FP/GP) visits				
Mean (SD)	6.87 ± 8.85	5.24 ± 6.45	0.21	< 0.001
Median (IQR)	5.00 (2.00–8.00)	4.00 (2.00–7.00)	0.21	< 0.001
Endocrinologist visits				
Mean (SD)	0.23 ± 0.93	0.06 ± 0.48	0.22	< 0.001
Internal medicine visits				
Mean (SD)	1.03 ± 2.90	0.62 ± 2.36	0.15	< 0.001
**Laboratory measurements**				
HbA1c	1083 (37.7%)	929,242 (13.8%)	0.57	< 0.001
HbA1c > 8.5%	397 (13.8%)	100,258 (1.5%)	0.48	< 0.001
Mean (SD)	7.88 ± 2.29	6.63 ± 1.37	0.67	< 0.001
Median (IQR)	7.30 (6.00–9.40)	6.20 (5.80–7.10)	0.53	< 0.001
LDL cholesterol	485 (16.9%)	955,143 (14.2%)	0.08	< 0.001
Mean (SD) mmol/L	2.52 ± 1.49	2.71 ± 1.02	0.15	< 0.001
Median (IQR) mmol/L	2.31 (1.49–3.26)	2.58 (1.93–3.38)	0.25	< 0.001
HDL cholesterol	1202 (41.9%)	986,111 (14.6%)	0.64	< 0.001
Mean (SD) mmol/L	0.97 ± 0.38	1.27 ± 0.38	0.79	< 0.001
Median (IQR) mmol/L	0.91 (0.76–1.11)	1.21 (1.00–1.47)	0.93	< 0.001
non-HDL cholesterol	327 (11.4%)	18,595 (0.3%)	0.49	< 0.001
Mean (SD) mmol/L	5.14 ± 2.27	4.58 ± 1.38	0.30	< 0.001
Median (IQR) mmol/L	4.68 (3.75–5.98)	4.52 (3.64–5.40)	0.17	0.001
Total cholesterol	1206 (42.0%)	994,409 (14.7%)	0.63	< 0.001
Mean (SD) mmol/L	6.41 ± 2.75	4.78 ± 1.25	0.76	< 0.001
Median (IQR) mmol/L	5.79 (4.71–7.33)	4.66 (3.85–5.57)	0.74	< 0.001
ALT	1282 (44.7%)	1,384,012 (20.5%)	0.53	< 0.001
Mean (SD) U/L	40.99 ± 39.83	27.36 ± 29.50	0.39	< 0.001
Median (IQR)	31.00 (21.00–46.00)	22.00 (17.00–31.00)	0.58	< 0.001
Corrected calcium	210 (7.3%)	166,005 (2.5%)	0.23	< 0.001
Mean (SD) mmol/L	2.30 ± 0.15	2.31 ± 0.11	0.12	0.045
Median (IQR) mmol/L	2.29 (2.23–2.37)	2.31 (2.24–2.37)	0.10	0.127

Standardized difference > 10% are considered statistically significant; < 1% of income quintiles were missing and were re-coded as ‘3’; Small cells (< 6) are suppressed as per ICES privacy policy; Comorbidities were obtained in the 5 years prior to the index date; Health care utilization measures were obtained in the 1 year prior to index date; Health care utilization measures were obtained in the 1 year prior to index date; Laboratory measurements were obtained in the 1 year prior to index prescription date; Evidence of pregnancy in women was obtained in the 1 year prior and within 9 month after to index date. Please refer to supplementary Table 1 for further information on coded data included for each variable. ^a^Missing income (< 1%) was recoded as ‘3’. Missing Charlson (~ 50%) was due to patients having no hospitalizations for relevant comorbidities found during the 5 year lookback period; they were recoded as ‘0’; ^*P*-value for variable as a whole

*Abbreviations*: *VS-HTG* very severe hypertriglyceridemia (TG > 20 mmol/L), *yrs.* years, *SD* Standard deviation, *IQR* Interquartile range, *GP* General Practitioner, *HTN* hypertension, *CAD* coronary artery disease, *PVD* peripheral vascular disease, *CVD* cerebrovascular disease, *CKD* chronic kidney disease, *FP* family practitioner, *HbA1c* hemoglobin A1c, *LDL* low-density lipoprotein, *HDL* high density lipoprotein, *ALT* alanine aminotransferase

**Fig. 2:**
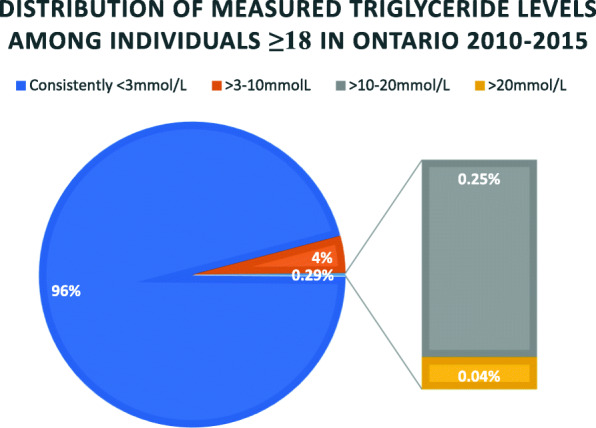
Summary of measured triglyceride (TG) values in Ontario from 2010 to 2015. Among 7,040,865 individuals ≥18 years with measured TG in Ontario between 2010 and 2015: 6,742,506 (96%) had no TG value measured above 3 mmol/L; 21,484 individuals in Ontario (0.29%) had at least one TG value > 10 mmol/L, with 0.25% (*n* = 17,615) having severe hypertriglyceridemia (TG > 10–20 mmol/L) and 0.04% (n = 2869) having very severe hypertriglyceridemia (TG > 10 mmol/L). 277,875 (4%) individuals were not included in the other three cohorts and were assumed to have at least one measured TG value within the range of > 3–10 mmol/L

Incidence of S-HTG and VS-HTG in Ontario adults between 2010 and 2015 was 163.61 and 26.65 per 100,000 (~ 1 in 615 and 1 in 3750) adults overall, and 250.18 and 40.75 per 100,000 (~ 1 in 400 and 1 in 2500) adults who had at least one TG test (Table 2). Combined incidence rate of TG > 10 mmol/L was ~ 1 in 344 adults who had at least one TG value and ~ 1 in 526 adults overall (Fig. 2). The highest incidence of S-HTG and VS-HTG appeared between ages 31–45 (315.17 and 63.07/100,000 with a TG test; 197.12 and 39.45/100,000 population) and 46–65 (324.86 and 48.21/100,000 with a TG test; 266.40 and 39.54/100,000 population) (Table 2). Overall, incidence was higher in men (Supplemental Table 3).

**Table 2:** Incidence of severe (S-HTG; TG > 10-20 mmol/L) and very severe (VS-HTG; TG > 20 mmol/L) hypertriglyceridemia in Ontario by age group

	Ages 18–30	Ages 31–45	Ages 46–65	Ages 66+	Total
Number of individuals with at least one TG test	761,224	1,709,237	3,071,797	1,498,607	7,040,865
Mean Ontario population between 2010 and 2015	2,412,041	2,732,915	3,745,940	1,875,874	10,766,770
S-HTG					
n	835	5387	9979	1414	17,615
Incidence in those with at least one TG test (per 100,000)	109.69	315.17	324.86	94.35	250.18
Incidence in Ontario population (per 100,000)	34.62	197.12	266.40	75.38	163.61
VS-HTG					
n	174	1078	1481	136	2869
Incidence in those with at least one TG test (per 100,000)	22.86	63.07	48.21	9.08	40.75
Incidence in Ontario population (per 100,000)	7.21	39.45	39.54	7.25	26.65

*Abbreviations*: *S-HTG* severe hypertriglyceridemia (TG > 10–20 mmol/L), *VS-HTG* very severe hypertriglyceridemia (TG > 20 mmol/L), *ON* Ontario, *pop* population, *n* number of individuals

Medication use was only available for individuals ≥65 years (*N* = 136 in the VS-HTG cohort) (Supplemental Table 4). At baseline, a higher proportion of VS-HTG patients used a fibrate (18.4% vs 1.6%; SD 0.58) and a lower proportion used statins (40.4% vs 45.7%; StDiff 0.11) compared to the comparison cohort (*N* = 1,392,795). A higher proportion of VS-HTG patients also appeared to use other lipid-lowering agents (11.8% vs 4.2%; StDiff 0.28), oral furosemide (16.2% vs 6.3%; StDiff 0.32) chlorthalidone (< 5% vs 0.6%; StDiff 0.14) and salicylates (5.9% vs 3.5%; StDiff 0.11) (supplemental Table 3).

Results of univariable and multivariable analysis are presented in Table 3. In multivariable analysis, significant predictors of VS-HTG were diabetes (OR 5.38, CI 4.93–5.88), male sex (OR 3.83, CI 3.50–4.18), alcohol abuse (OR 2.47, CI 1.90–3.19), chronic liver disease (OR 1.71, CI 1.48–1.97), hypertension (OR 1.69, CI 1.54–1.86), obesity (OR 1.49, CI 1.13–1.98), and chronic kidney disease (OR 1.39, CI 1.19–1.63). Older age was associated with reduced risk of VS-HTG (OR 0.64/decade, CI 0.62–0.66). Higher income quintiles (4 and 5) were associated with lower odds of having VS-HTG in both univariable and multivariable analysis (0.78, CI 0.70–0.88 for quintile 4 and 0.72, CI 0.63–0.81 for quintile 5). In univariable analysis**,** lowest income quintile was associated with VS-HTG (OR 1.21, CI 1.09–1.35) but this did not remain significant in the multivariate analysis. Likewise, higher Charlson comorbidity scores (moderate to high) were associated with VS-HTG in the univariate analysis. A Charlson index of 3+ was no longer significantly associated with VS-HTG in multivariable analysis (Table 3).

**Table 3:** Predictors of VS-HTG, ranked by odds ratio

	Univariable analysis				Multivariable analysis			
	Odds Ratio	LCL	UCL	***P***-value	Odds Ratio	LCL	UCL	***P***-value
Diabetes	4.85	4.50	5.22	< 0.0001	5.38	4.93	5.88	< 0.0001
Sex								
Male	4.24	3.88	4.63	< 0.0001	3.83	3.50	4.18	< 0.0001
Female	REF	REF	REF		REF	REF	REF	
Alcohol	6.34	4.96	8.09	< 0.0001	2.47	1.90	3.19	< 0.0001
Chronic liver disease	2.42	2.11	2.77	< 0.0001	1.71	1.48	1.97	< 0.0001
HTN	1.55	1.44	1.67	< 0.0001	1.69	1.54	1.86	< 0.0001
Obesity	3.27	2.49	4.29	< 0.0001	1.49	1.13	1.98	0.0049
CKD	2.33	2.01	2.69	< 0.0001	1.39	1.19	1.63	< 0.0001
Income quintile								
1 - lowest	1.21	1.09	1.35	0.0005	1.09	0.98	1.22	0.1134
2	1.06	0.95	1.18	0.3011	1.02	0.92	1.14	0.6747
3	REF	REF	REF		REF	REF	REF	
4	0.74	0.66	0.84	< 0.0001	0.78	0.70	0.88	< 0.0001
5 - highest	0.63	0.55	0.71	< 0.0001	0.72	0.63	0.81	< 0.0001
Age (per decade)	0.84	0.82	0.86	< 0.0001	0.64	0.62	0.66	< 0.0001
Charlson comorbidity index^a^								
0	REF	REF	REF		REF	REF	REF	
1	2.98	2.66	3.34	< 0.0001	1.61	1.42	1.82	< 0.0001
2	2.00	1.69	2.37	< 0.0001	1.26	1.05	1.50	0.0137
3+	2.99	2.54	3.51	< 0.0001	1.15	0.95	1.38	0.1504

Sample size is the full cohort (*N* = 6,745,375); Exposed (*n* = 2869); Unexposed (*n* = 6,742,506) All predictors were associated with the odds of having hypertriglyceridemia. Diabetes includes both type 1 and type 2

*Abbreviations*: *LCL* lower confidence limit, *UCL* upper confidence limit, *HTN* hypertension, *CKD* chronic kidney disease, *REF* used as reference

^a^ Missing income (< 1%) was recoded as ‘3’. Missing charlson (~ 50%) was due to patients having no hospitalizations for relevant comorbidities found during the 5 year lookback period; they were recoded as ‘0’

### Outcomes

The majority of individuals with VS-HTG received follow-up healthcare within 2 years of their TG test (Table 4); 98.4% had at least one follow up visit to a family physician (FP), 32.8% had at least one visit to an endocrinologist and 56.7% had at least one visit to a general internist. The majority achieved TG reduction below the high-risk pancreatitis threshold of 10 mmol/L, with 89.3% having a repeat TG test. The last TG measured had a median TG value of 4.7 mmol/L (IQR 2.7–9.1) and lowest TG value had a median TG value of 3.3 mmol/L (IQR 2.0–5.9). For individuals > 65 years of age, new prescriptions for statins were provided in 32.4% and for fibrates, in 19.9% (Supplemental Table 3).

**Table 4:** Healthcare Patterns and Events of Interest in patients with VS-HTG

	Within 1 year	Within 2 years
**Healthcare patterns (*N*** **= 2869)**		
General practitioner visit	2786 (97.1%)	2822 (98.4%)
Endocrinologist visit	775 (27.0%)	941 (32.8%)
Internist visit^a^	1279 (44.6%)	1628 (56.7%)
TG test	2218 (77.3%)	2561 (89.3%)
Last TG value		
Mean (SD) (mmol/L)	7.66 ± 8.29	7.68 ± 8.35
Median (IQR) (mmol/L)	4.7 (2.7–9.1)	4.7 (2.7–9.1)
Lowest TG value		
Mean (SD) (mmol/L)	6.09 ± 6.74	5.30 ± 6.05
Median (IQR) (mmol/L)	3.8 (2.2–7.1)	3.3 (2.0–5.9)
**New prescriptions (restricted to ages 66+,*N*** **= 136)**		
Statin	35 (25.7%)	44 (32.4%)
Fibrate	25 (18.4%)	27 (19.9%)
Niacin	0	0
**Events**	Within 1 year	Within 2 years
At least one hospital encounter for pancreatitis	118 (4.1%)	171 (6.0%)
At least one hospital encounter for acute myocardial infarction	22 (0.8%)	39 (1.4%)
At least one hospital encounter for Ischemic stroke	12 (0.4%)	24 (0.8%)

^a^ For the purposes of this data ‘internal medicine’ refers specifically to the subspecialty of general internal medicine and does not include other internal medicine subspecialties

Following detection of VS-HTG, pancreatitis occurred in 4.1 and 6.0% of individuals in the VS-HTG cohort within one and 2 years, respectively (Table 4). Within 2 years, 1.4 and 0.8% of individuals in the VS-HTG cohort had at least one hospital encounter for acute myocardial infarction and ischemic stroke, respectively (Table 4).

## Discussion

The incidence of TG > 10–20 (S-HTG) and > 20 mmol/L (VS-HTG) between 2010 and 2015 was approximately 1 in 400 (0.25%) and 1 in 2500 (0.04%) among adults in Ontario with measured TG, and 1 in 613 (0.16%) and 1 in 3750 (0.27%) of the adult population, respectively. These numbers align with prevalence rates reported in other studies [14, 16, 17]. Peak age of incidence was 30–65 years, which may correspond to the age at which a screening lipid profile may first be conducted, or the age at which related chronic conditions start to manifest. While the Endocrine Society defines severe hypertriglyceridemia as a serum TG level > 1000 mg/dL (11.3 mmol/L) and very severe as > 3000 mg/dL (22.6 mmol/L) [1], the approximations of 10 and 20 mmol/L, respectively, are more practical for a Canadian population.

Significant predictors of VS-HTG in Ontario included male sex, as well as known risk factors for HTG, including diabetes, chronic liver disease, alcohol abuse, obesity and chronic kidney disease. Hypertension was likely a predictor given its association with metabolic syndrome. The two most significant controllable predictors were diabetes and alcohol abuse. The definition used for alcohol abuse in this study was broad to allow for increased sensitivity, but included any complication that stemmed from chronic or acute alcohol ingestion (see Supplemental Table 1). It is thus difficult to determine which specific alcohol-related behaviors or patterns of intake may be most contributory.

The VS-HTG cohort had more contacts with healthcare providers, which may relate to more associated chronic diseases. This may have predisposed individuals to having had a screening lipid test. Patients in the VS-HTG group were also more likely to have had HbA1c testing, and to have an HbA1c > 8.5%, likely relating to the higher prevalence of metabolic syndrome and diabetes in the VS-HTG cohort. Mean LDL-C was minimally but significantly higher in the VS-HTG. Mean HDL-C was significantly lower in the VS-HTG cohort, reflecting the inverse relationship between TG and HDL-C. Non-HDL and total cholesterol were higher in the VS-HTG cohort, likely driven by elevated TG-rich lipoproteins and remnant particles.

While other studies showed poor follow up care following identification of HTG, in this Ontario cohort, no significant gaps in appropriate care were identified. This may be due to universal healthcare access in Ontario, which was not a feature of other populations studied [16]. Within the 2 year follow-up period, 98.4% of individuals in the VS-HTG cohort were seen by a FP and 89.3% had a repeat TG test. One-third were seen by an endocrinologist and over half by an internist. Follow-up TG tests showed significant reductions in TG levels, with the median TG level falling below the threshold for pancreatitis risk (4.7 mmol/L; IQR 2.7–9.1). The median of the lowest recorded TG values remained elevated at 3.3 mmol/L (IQR 2.0–5.9) (normal < 1.7 mmol/L), likely reflecting the limitations of currently available pharmacological and lifestyle interventions to fully correct TG to a normal range, particularly in those with underlying inherited metabolic defects. Discussions of current management recommendations for HTG and options are beyond the scope of this work, but are discussed in detail elsewhere [5]. In the subset of the VS-HTG cohort that were over the age of 65, most were on, or were placed on, a statin (40.4% at baseline, 32.4% with new prescriptions following identification of VS-HTG) and a third were on a fibrate (18.4% at baseline, 19.9% with new prescriptions). The substantial reduction in TG levels, despite only a third of patients with medication information available being placed on a fibrate, suggests that control of contributory secondary factors, such as diabetes, obesity or lifestyle may have played a considerable role in the improvements in TG levels.

Pancreatitis rates in individuals with VS-HTG (4.1% within 1 year, 6% within 2 years) were within the range of expectation based on other reports [27]. There was no apparent excess risk of atherosclerotic cardiovascular disease within the VS-HTG cohort at baseline or within the 2-year follow up period. However, this study was not designed to examine these outcomes. A separate study focusing on the risk of ASCVD and ischemic stroke in the VS-HTG population that accounts for potential confounders may provide further support for the findings observed.

Future studies that evaluate TG as a continuous variable would help define the risk of acute pancreatitis associated with a given degree of HTG. Further investigations that focus on outcomes in VS-HTG patients is another avenue of investigation, including prevalence and predictors of pancreatitis and its complications. Genetic investigation into a subset of this cohort may also help define the spectrum of genetic variation that may underlie a presentation of VS-HTG.

### Comparison with other provinces in Canada and European Union (EU) countries

All Canadian provinces have universal healthcare coverage, however there are differences across the Canadian provinces in terms of risk factors for VS-HTG (e.g. obesity and type 2 diabetes) For example, provinces such as Saskatchewan, New Brunswick, Nova Scotia and Newfoundland and Labrador have higher rates of obesity (35–38%) compared to Ontario (26%), which is just below the national average of 27% (range of all provinces 22–38%) [28]. Approximately 30% of Ontarians live with diabetes or prediabetes, which is slightly higher than the national average of 29% (range of all province 25–35%) [29]. There is also ethnic variation between the provinces, with Ontario and British Columbia having the highest rates of ethnic diversity (29.3 and 30.3% of adult population identifying as visible minorities, respectively, national average 22.3%, range for all provinces 2.3–30.3%) [30].

Most EU countries are similar to Ontario in that they have universal access to healthcare, but may differ in other ways that could affect prevalence and predictors of VS-HTG. Obesity rates are overall lower in the EU compared to Ontario (17%; range 10.7–30.6%) [31], as are rates of type 2 diabetes (~ 10%) [32]. There may also be differences in ethnic diversity.

### Study strengths and limitations

Strengths of this study include access to province-wide laboratory data, the large study cohort with access to universal health services and the use of well-defined coding algorithms used to investigate patient characteristics. It is also, to our knowledge, the first study to systematically assess incidence and characteristics associated with HTG in a Canadian population.

While this study provides useful province-wide information on incidence of S- and VS-HTG, there are some limitations. Prevalence was not examined and information was only gathered over a 5-year time frame with the study collection period ending in 2015, limiting capture of more recent trends. Furthermore, not all laboratory data were available for all individuals (e.g. HbA1c), potentially creating bias. Additionally, not all laboratories submitted data to OLIS simultaneously and data may be less complete for earlier time points [22]. Inclusion in the VS-HTG or S-HTG cohort were also based upon a single TG measurement, which could have allowed for inclusion of individuals in these cohorts who had only transiently elevated TG levels. However, TG levels in this range, even transiently can expose an individual to the risk of pancreatitis. Furthermore, literature supports day-to-day variability in TG of approximately 20% [33], therefore even accounting for maximal variability, TG levels are likely to remain elevated on subsequent tests. Information on pharmacological treatment was limited to a subset of the VS-HTG cohort, limiting the usefulness and generalizability of this data. The use of over the counter supplements could not be captured with our data sources. Certain susceptibility states, such as obesity and alcohol abuse, may be underestimated in the administrative data due to low coding sensitivity, which may have underestimated their contribution to HTG. Individuals with poor access to healthcare may be under-represented in the study sample as they would be less likely to obtain a lipid profile. Similarly, it is possible that including these patients with undetected VS-HTG may have resulted in lower follow-up rates than seen in this VS-HTG cohort. Additionally, data regarding visits to some specialists that may be involved in the management of VS-HTG, such as cardiologists or gastroenterologists, was not captured. While it was concluded that there was no significant care gap, it could be argued that specialist referral would be most appropriate for anyone with a history of VS-HTG, given it is a rare condition with potentially serious side effects, suggesting that there may still be a gap in appropriate care within Ontario. There were overall low numbers in the VS-HTG cohort, but only 10 predictors were chosen and there should have had sufficient statistical power. Additionally, the baseline rates of pancreatitis and use of fibrates were high in those with VS-HTG, suggesting that these may not have all been incident cases. Finally, given the observational nature of the data set, causality cannot be determined, and the findings may not be generalizable outside Ontario.

## Conclusions

In conclusion, this study shows that ~ 1/400 adults in Ontario have S-HTG and ~ 1/2500 had new evidence of VS-HTG from 2010 to 2015. Peak incidence occurs between the ages of 30 and 65 years. Conditions that are most strongly associated with VS-HTG include diabetes, male sex, alcohol, chronic liver disease, hypertension, obesity and chronic kidney disease. No significant care gap was identified for individuals in Ontario with identified VS-HTG and the majority had repeat TG below the threshold for pancreatitis risk.

These findings may assist clinicians in recognizing individuals at heightened risk for VS-HTG, who may benefit from increased surveillance. Male patients with diabetes, obesity and alcohol abuse are at the highest risk; early attention to these patients may assist in developing an individualized treatment plan to monitor for HTG and prevent associated adverse outcomes.

## Supplementary Information

**Additional file 1: Supplemental Table 1.** Databases Utilized. **Supplemental Table 2.** RECORD checklist (from (17)). **Supplementary Table 3.** Incidence of severe (S-HTG; TG >10-20mmol/L) and very severe (VS-HTG; TG>20mmol/L) hypertriglyceridemia in Ontario by age group and gender. **Supplemental Table 4.** Prescription characteristics of VS-HTG and comparison cohorts

## Data Availability

The analysis was conducted by members of the ICES Western facility (London, Ontario). Alexandra Ouédraogo is responsible for the data analysis. The protocol can be obtained by emailing Dr. Kristin Clemens at Kristin.Clemens@sjhc.london.on.ca.

## Abbreviations

S-HTG: Severe hypertriglyceridemia
VS-HTG: Very severe hypertriglyceridemia
TG: Triglyceride
OR: Odds ratio
SD: Standard deviation
LPL: Lipoprotein lipase
APOC2: Apolipoprotein C-II
APOA5: Apolipoprotein A-V
LMF1: Lipase maturation factor 1
GPIHBP1: Glycosylphosphatidylinositol-anchored high density lipoprotein binding protein 1
OHIP: Ontario Health Insurance Program
RPDB: Registered Persons Database
CAPE: Client Agency Program Enrolment
CIHI-DAD: Canadian Institutes for Health Information’s Discharge Abstract Database
NACRS: National Ambulatory Care Reporting System
ICD-10: International Classification of Diseases 10th Revision (ICD-10)
CCI: Canadian Classification of Health Interventions
HYPER: Hypertension database
ODD: Ontario Diabetes Database
ODB: Ontario Drug Benefit
DIN: Drug Identification Number
IQR: Interquartile range
StDiff: Standardized differences
CI: Confidence intervals
HTN: Hypertension
FP: Family physician
