# Supplementary material for: Incidence, predictors and patterns of care of patients with very severe hypertriglyceridemia in Ontario, Canada: a population-based cohort study
Source: Lipids Health Dis. 2021 Sep 3;20:98. doi: 10.1186/s12944-021-01517-6 (PMC8417954; doi:10.1186/s12944-021-01517-6)
Supplement: Supplementary file 1 — Additional file 1: Supplemental Table 1. Databases Utilized. Supplemental Table 2. RECORD checklist (from (17)). Supplementary Table 3. Incidence of severe (S-HTG; TG >10-20mmol/L) and very severe (VS-HTG; TG>20mmol/L) hypertriglyceridemia in Ontario by age group and gender. Supplemental Table 4. Prescription characteristics of VS-HTG and comparison cohorts [file 12944_2021_1517_MOESM1_ESM.docx]

**Appendix:**

**Supplemental Table 1: Databases Utilized**

| **Variable** | **Database** | **Codes** |
| --- | --- | --- |
| Age | RPDB |  |
| Sex | RPDB |  |
| Income quintile | RPDB |  |
| Rostered to family doctor | CAPE |  |
| Charlson comorbidity status | CIHI-DAD |  |
| Coronary artery disease (excluding angina) | CIHI-DAD  NACRS  OHIP | ICD10: "I21", "I22", "Z955", "T822", "I25"  CCI: "1IJ50", "1IJ76"  OHIP fee codes: "R741", "R742", "R743", "G298", "E646", "E651", "E652", "E654", "E655", "Z434", "Z448"  OHIP Dx codes: "410", "412" |
| Cerebrovascular disease | CIHI-DAD  NACRS | ICD10: "I60", "I600", "I601", "I602", "I603", "I604", "I605", "I606", "I607", "I608", "I609", "I61", "I610", "I611", "I612", "I613", "I614", "I615", "I616", "I618", "I619", "I630", "I631", "I632", "I633", "I634", "I635", "I638", "I639", "I64", "H341", "G450", "G451","G452","G453","G458","G459","H340"  OHIP Dx codes: "436", "432", "435" |
| Diabetes | ODD (ICES validated cohort) | Lorraine L. Lipscombe, Jeremiah Hwee, Lauren Webster, Baiju R. Shah, Gillian L. Booth and Karen Tu. Identifying diabetes cases from administrative data: a population-based validation study. BMC Health Services |
| Peripheral vascular disease | CIHI-DAD  OHIP | ICD10: "I700", "I702", "I708", "I709", "I731", "I738", "I739", "K551"  CCI: "1KA76", "1KA50", "1KE76", "1KG50", "1KG57", "1KG76MI", "1KG87", "1IA87LA", "1IB87LA", "1IC87LA", "1ID87", "1KA87LA", "1KE57"  OHIP fee codes: "R787", "R780", "R797", "R804", "R809", "R875", "R815", "R936", "R783", "R784","R785", "E626", "R814", "R786", "R937", "R860", "R861", "R855", "R856", "R933", "R934", "R791", "E672", "R794", "R813", "R867", "E649" |
| Chronic kidney disease | CIHI-DAD  OHIP | ICD10: "E102", "E112", "E132", "E142", "I12", "I13", "N00", "N01", "N02", "N03", "N04", "N05", "N06", "N07", "N08", "N10", "N11", "N12", "N13", "N14", "N15", "N16", "N17", "N18", "N19", "N20", "N21", "N22", "N23"  OHIP Dx codes: "403", "585" |
| Pancreatitis | CIHI-DAD | ICD10: "K85", "B252", "B263", "K860", "K861" |
| Hypertension | HYPER (ICES validated cohort) | Tu K, Chen Z, Lipscombe LL, Canadian Hypertension Education Program Outcomes Research Taskforce. Prevalence and incidence of hypertension from 1995 to 2005: a population-based study. Canadian Medical Association Journal. 2008 May 20;178(11):1429-35. |
| Chronic liver disease | CIHI-DAD  NACRS  OHIP | ICD10: "B16", "B17", "B18", "B19", "I85", "R17", "R18", "R160", "R162", "B942", "Z225", "E831", "E830", "K70", "K713", "K714", "K715", "K717", "K721", "K729", "K73", "K74", "K753", "K754", "K758", "K759", "K76", "K77"  OHIP Dx codes: "571", "573", "070"  OHIP fee codes: "Z551", "Z554" |
| Alcohol use | CIHI-DAD | ICD10: "E244", "E512", "E52", "F10", "G312", "G621", "G721", "I426", "K292", "K70", "K860", "T51", "X45", "X65", "Y15", "Y573", "Z502", "Z714", "Z721" |
| Hypothyroidism | CIHI-DAD | ICD10: "E00", "E01", "E02", "E03", "E890" |
| Multiple myeloma | CIHI-DAD | ICD10: "C900" |
| Obesity | CIHI-DAD | ICD10: "E66.25", "E66.26", "E66.27", "E66.28", "E66.29", "E66.0", "E66.1", "E66.2", "E66.8", "E66.9", "E66.2”,"E66.2" |
| Pregnancy | CIHI-DAD | ICD10: “Z34", P95", "Z371", "Z373", "Z374", "O00", "O021", "O03", "O04", "O08", "O60", "O42", "P072", "P073"  CCI: "5CA88", "5CA20FK", "5CA24", "5MD5", "5MD6", "5MD4"  OHIP FEE: "A922", "A920", "P001", "S752", "S785", "S756", "S768", "S784", "S770", "P006", "P007", "P008", "P009", "P010", "P011", "P013", "P014", "P014", "P015", "P016", "P016", "P018", "P020", "P022", "P023", "P027", "P028", "P029", "P030", "P031", "P032", "P034", "P036", "P038", "P039", "P041", "P042", "P045", "P046"  OHIP DX: "632", "633", "634", "640" |
| Gallstone disease | CIHI-DAD | ICD10: "K80", "K81", "K82", "K83", "K85" |
| Acute myocardial infarction | CIHI-DAD | ICD10: "I21", "I22" |
| Nephrotic Syndrome | CIHI-DAD | ICD 10: "N044", "N022", "N043", "N040", "N08", "N048", "N049", "N033", "N052" |
| Ischemic Stroke: | CIHI-DAD | ICD-10: "I63", "I64", "I65", "I66", "I67", "I68 |
| GP/FP visit | OHIP  IPDB | OHIP spec: “00”  IPDB Mainspecialty: “GP/FP” |
| Internist visit | OHIP  IPDB | OHIP spec: “13”  IPDB Mainspecialty: “INTERNAL MEDICINE” |
| Endocrinologist visit | OHIP  IPDB | OHIP spec: “15”  IPDB Mainspecialty: “ENDOCRINOLOGY” |
| HbA1c | OLIS | LOINC: “17855-8”, “17856-6“, “41995-2“, “4548-4”, “59261-8”, “71875-9” |
| LDL-C | OLIS | LOINC: “22748-8“, “39469-2“ |
| HDL-C | OLIS | LOINC: “14646-4“, “32309-7 |
| Non-HDL | OLIS | LOINC: “70204-3“ |
| Total Cholesterol | OLIS | LOINC: “14647-2“ |
| Corrected calcium | OLIS | LOINC: “29265-6”, “2000-8”, “1751-7” |
| ALT | OLIS | LOINC: “1742-6“, “1743-4“, “1744-2“ |
| Lipid medication | ODB |  |
| Triglyceride value | OLIS | LOINC: “14927-8“, “47210-0“ |

Abbreviations: RPDB**:** registered persons database; CAPE: Client Agency Program Enrolment database: CIHI-DAD: Canadian Institutes for Health Information’s Discharge Abstract Database; NARCRS: National Ambulatory Care Reporting System; OHIP: Ontario Health Insurance Program; ICD-10: the International Classification of Diseases 10^th^ Revision; CCI: Canadian Classification of Health Interventions; HYPER: hypertension database; ODD: Ontario Diabetes Database; ODB: Ontario Drug Benefit (ODB) database. OLIS: Ontario Laboratories Information System (OLIS).

**Supplemental Table 2: RECORD checklist (from (17))**

|  | **Item No** | **Recommendation** | **Reported** |
| --- | --- | --- | --- |
| Title and abstract | 1 | 1.1 The type of data used should be specified in the title or abstract. When possible, the name of the databases should be included. | Abstract |
|  |  | 1.2 If applicable, the geographic region and time frame within which the study took place should be reported in the title or abstract. | Abstract |
|  |  | 1.3 If linkage between databases was conducted for the study, this should be clearly stated in the title or abstract | Abstract |
| Introduction | | |  |
| Background/rationale | 2 | Explain the scientific background and rationale for the investigation being reported | Introduction |
| Objectives | 3 | State specific objectives, including any pre-specified hypotheses | Introduction |
| Methods | | |  |
| Study design | 4 | Present key elements of study design early in the paper | Methods |
| Setting | 5 | Describe the setting, locations, and relevant dates, including periods of recruitment, exposure, follow-up, and data collection | Methods |
| Participants | 6 | 6.1 The methods of study population selection should be listed in detail. If this is not possible, an explanation should be provided. | Methods |
|  |  | 6.2 Any validation studies of the codes or algorithms used to select the population should be referenced. If validation was conducted for this study and not published elsewhere, detailed methods and results should be provided. | Methods |
|  |  | 6.3 If the study involved linkage of databases, consider use of a flow diagram or other graphical display to demonstrate the linkage process, including the number of individuals with linked data at each stage. | Figure 1 |
| Variables | 7 | A complete list of codes and algorithms used to classify exposures, outcomes, confounders, and effect modifiers should be provided. If these cannot be reported, an explanation should be provided. | Supplemental table 1 |
| Data sources/ measurement | 8 | For each variable of interest, give sources of data and details of methods of assessment (measurement). Describe comparability of assessment methods if there is more than one group | Methods, Supplemental table 1 |
| Bias | 9 | Describe any efforts to address potential sources of bias | Methods |
| Study size | 10 | Explain how the study size was arrived at | Methods |
| Quantitative variables | 11 | Explain how quantitative variables were handled in the analyses. If applicable, describe which groupings were chosen and why | Methods |
| Statistical methods | 12 | 12.1 Describe all statistical methods, including those used to control for confounding | Methods |
|  |  | 12.2 Describe any methods used to examine subgroups and interactions | Methods |
|  |  | 12.3 Explain how missing data were addressed | Not applicable |
|  |  | 12.4 If applicable, explain how loss to follow-up was addressed | Not applicable |
|  |  | 12.5 Describe any sensitivity analyses | Not applicable |
| Data access and cleaning methods |  | 12.6 Authors should describe the extent to which the investigators had access to the database population used to create the study population. | Methods |
|  |  | 12.7 Authors should provide information on the data cleaning methods used in the study | Methods |
| Linkage |  | 12.8 State whether the study included person-level, institutional-level, or other data linkage across two or more databases. The methods of linkage and methods of linkage quality evaluation should be provided. | Methods |
| Results | | |  |
| Participants | 13 | 13.1 Describe in detail the selection of the persons included in the study (i.e. study population selection), including filtering based on data quality, data availability, and linkage. The selection of included persons can be described in the text and/or by means of the study flow diagram. | Results, Figure 1 |
| Descriptive data | 14 | 14.1 Give characteristics of study participants (e.g. demographic, clinical, social) and information on exposures and potential confounders | Results, Tables 1,2 |
|  |  | 14.2 Indicate number of participants with missing data for each variable of interest | Results, Figure 1 |
|  |  | 14.3 Summarize follow-up time (e.g. average and total amount) | Results, Table 4 |
| Outcome data | 15 | Report numbers of outcome events or summary measures over time | Results, Figure 2, Table 1,4 |
| Main results | 16 | 16.1 Give unadjusted estimates and, if applicable, confounder-adjusted estimates and their precision (e.g. 95% confidence interval). Make clear which confounders were adjusted for and why they were included | Results, Table 3 |
|  |  | 16.2 Report category boundaries when continuous variables were categorized | Tables 2-4 |
|  |  | 16.3 If relevant, consider translating estimates of relative risk into absolute risk for a meaningful time period | Not applicable |
| Other analyses | 17 | Report other analyses done—e.g. analyses of subgroups and interactions, and sensitivity analyses | Results, Table 3 |
| Discussion | | |  |
| Key results | 18 | Summarize key results with reference to study objectives | Discussion |
| Limitations | 19 | Discuss the implications of using data that were not created or collected to answer the specific research question(s). Include discussion of misclassification bias, unmeasured confounding, missing data and changing eligibility over time, as they pertain to the study being reported. | Discussion |
| Interpretation | 20 | Give a cautious overall interpretation of results considering objectives, limitations, multiplicity of analyses, results from similar studies, and other relevant evidence | Discussion |
| Generalizability | 21 | Discuss the generalizability (external validity) of the study results | Discussion |
| Other information | | |  |
| Funding | 22 | 22.1 Give the source of funding and the role of the funders for the present study and, if applicable, for the original study on which the present article is based | Declarations |
| Accessibility of protocol, raw data and programming code |  | 22.2 Authors should provide information on how to access any supplemental information such as the study protocol, raw data, or programming code. | The dataset from this study is held securely in coded form at the Institute for Clinical Evaluative Sciences (ICES). While data sharing agreements prohibit ICES from making the dataset publicly available, access may be granted to those who meet pre-specified criteria for confidential access, available at www.ices.on.ca/DAS. The full dataset creation plan and underlying analytic code are available from the authors upon request, understanding that the programs may rely upon coding templates or macros that are unique to ICES. |

*Reference: Benchimol EI, Smeeth L, Guttmann A, Harron K, Moher D, Petersen I, et al. The REporting of studies Conducted using Observational Routinely-collected health Data (RECORD) statement. PLoS Med. 2015;12(10):e1001885.

*Checklist is protected under Creative Commons Attribution ([CC BY](http://creativecommons.org/licenses/by/4.0/)) license.

**Supplementary Table 3:** Incidence of severe (S-HTG; TG >10-20mmol/L) and very severe (VS-HTG; TG>20mmol/L) hypertriglyceridemia in Ontario by age group and gender

|  | **Ages 18-30** | **Ages 31-45** | **Ages 46-65** | **Ages 66+** | **Total** |
| --- | --- | --- | --- | --- | --- |
| ***Women*** | | | | | |
| Number of individuals with at least one TG test | 424,465 | 933,287 | 1,604,830 | 817,872 | 3,780,454 |
| Mean Ontario population between 2010-2015 | 1,201,563 | 1,392,309 | 1,895,645 | 1,047,061 | 5,536,578 |
| S-HTG |  |  |  |  |  |
| n | 206 | 1043 | 2387 | 564 | 4,200 |
| Incidence in those with at least one TG test (per 100,000) | 49 | 112 | 149 | 69 | 111 |
| Incidence in Ontario population (per 100,000) | 17 | 75 | 126 | 54 | 76 |
| VS-HTG |  |  |  |  |  |
| n | 39 | 211 | 322 | 58 | 630 |
| Incidence in those with at least one TG test (per 100,000) | 9.19 | 22.61 | 20.06 | 7.09 | 16.66 |
| Incidence in Ontario population (per 100,000) | 3.25 | 15.15 | 16.99 | 5.54 | 11.38 |
| ***Men*** | | | | | |
| Number of individuals with at least one TG test | 336,759 | 775,950 | 1,466,967 | 680,735 | 3,260,411 |
| Mean Ontario population between 2010-2015 | 1,210,478 | 1,340,606 | 1,850,295 | 828,813 | 5,230,192 |
| S-HTG |  |  |  |  |  |
| n | 629 | 4,344 | 7,592 | 850 | 13,415 |
| Incidence in those with at least one TG test (per 100,000) | 187 | 560 | 518 | 125 | 411 |
| Incidence in Ontario population (per 100,000) | 52 | 324 | 410 | 103 | 256 |
| VS-HTG |  |  |  |  |  |
| n | 135 | 867 | 1,159 | 78 | 2,239 |
| Incidence in those with at least one TG test (per 100,000) | 40.09 | 111.73 | 79.01 | 11.46 | 68.67 |
| Incidence in Ontario population (per 100,000) | 11.15 | 64.67 | 62.64 | 9.41 | 42.81 |

Abbreviations: S-HTG: severe hypertriglyceridemia (TG >10-20 mmol/L); VS-HTG: very severe hypertriglyceridemia (TG >20mmol/L); ON: Ontario; pop: population; n: number of individuals;

**Supplemental Table 4:** Prescription characteristics of VS-HTG and comparison cohorts

| **Characteristics in ages 66+** | **VS-HTG cohort**  **N=136** | **Comparison cohort**  **N=1,392,795** |  |
| --- | --- | --- | --- |
| Oral glucocorticoid | <6 | 8,741 (0.6%) | 0.01 |
| Statin | 55 (40.4%) | 636,487 (45.7%) | 0.11 |
| Fibrate | 25 (18.4%) | 22,422 (1.6%) | 0.58 |
| Niacin | 0 (0.0%) | 147 (0.0%) | 0.01 |
| Other lipid | 16 (11.8%) | 57,839 (4.2%) | 0.28 |
| Lipid combination (e.g. with blood pressure medication) | 0 | 13,984 (1.0%) | 0.14 |
| Metronidazole | <6 | 15,899 (1.1%) | 0.03 |
| Tetracycline | 0 (0.0%) | 2,287 (0.2%) | 0.06 |
| Oral furosemide | 22 (16.2%) | 87,477 (6.3%) | 0.32 |
| Hydrochlorothiazide | 32 (23.5%) | 315,376 (22.6%) | 0.02 |
| Chlorthalidone | <6 | 8,525 (0.6%) | 0.14 |
| Indapamide | <6 | 47,567 (3.4%) | 0.01 |
| Sulphasalazine | 0 | 13 (0.0%) | 0 |
| Azathioprine | 0 | 1,912 (0.1%) | 0.05 |
| Valproic acid | 0 | 908 (0.1%) | 0.04 |
| Sulindac | 0 | 2,665 (0.2%) | 0.06 |
| Salicylates | 8 (5.9%) | 48,599 (3.5%) | 0.11 |
| HIV/AIDS specific meds | 0 | 128 (0.0%) | 0.01 |
| ACE/ARB | 38 (27.9%) | 396,107 (28.4%) | 0.01 |
| Beta blocker | 54 (39.7%) | 320,828 (23.0%) | 0.37 |

Standardized difference > 10% are considered statistically significant; <1% of income quintiles were missing and were re-coded as ‘3’; Small cells (<6) are suppressed as per ICES privacy policy; Medication use were obtained in the 1 year prior to index date; A few other medications of interest (e.g. oral estrogen, pentamidine, 5-ASA, L-asparaginase, didanosine) were not found.
